# Complete genome sequence of *Halogeometricum borinquense* type strain (PR3^T^)

**DOI:** 10.4056/sigs.23264

**Published:** 2009-09-24

**Authors:** Stephanie Malfatti, Brian J. Tindall, Susanne Schneider, Regine Fähnrich, Alla Lapidus, Kurt LaButtii, Alex Copeland, Tijana Glavina Del Rio, Matt Nolan, Feng Chen, Susan Lucas, Hope Tice, Jan-Fang Cheng, David Bruce, Lynne Goodwin, Sam Pitluck, Iain Anderson, Amrita Pati, Natalia Ivanova, Konstantinos Mavromatis, Amy Chen, Krishna Palaniappan, Patrik D’haeseleer, Markus Göker, Jim Bristow, Jonathan A. Eisen, Victor Markowitz, Philip Hugenholtz, Nikos C. Kyrpides, Hans-Peter Klenk, Patrick Chain

**Affiliations:** 1 DOE Joint Genome Institute, Walnut Creek, California, USA; 2 Lawrence Livermore National Laboratory, Livermore, California, USA; 3 DSMZ - German Collection of Microorganisms and Cell Cultures GmbH, Braunschweig, Germany; 4 Los Alamos National Laboratory, Bioscience Division, Los Alamos, New Mexico, USA; 5 Biological Data Management and Technology Center, Lawrence Berkeley National Laboratory, Berkeley, California, USA; 6 University of California Davis Genome Center, Davis, California, USA

**Keywords:** halophile, free-living, non-pathogenic, aerobic, pleomorphic cells, euryarchaeon

## Abstract

*Halogeometricum borinquense* Montalvo-Rodríguez *et al*. 1998 is the type species of the genus, and is of phylogenetic interest because of its distinct location between the *halobacterial* genera *Haloquadratum* and *Halosarcina. H. borinquense* requires extremely high salt (NaCl) concentrations for growth. It can not only grow aerobically but also anaerobically using nitrate as electron acceptor. The strain described in this report is a free-living, motile, pleomorphic, euryarchaeon, which was originally isolated from the solar salterns of Cabo Rojo, Puerto Rico. Here we describe the features of this organism, together with the complete genome sequence, and annotation. This is the first complete genome sequence of the halobacterial genus *Halogeometricum*, and this 3,944,467 bp long six replicon genome with its 3937 protein-coding and 57 RNA genes is part of the *** G****enomic* *** E****ncyclopedia of* *** B****acteria and* *** A****rchaea * project.

## Introduction

**Figure 1:**
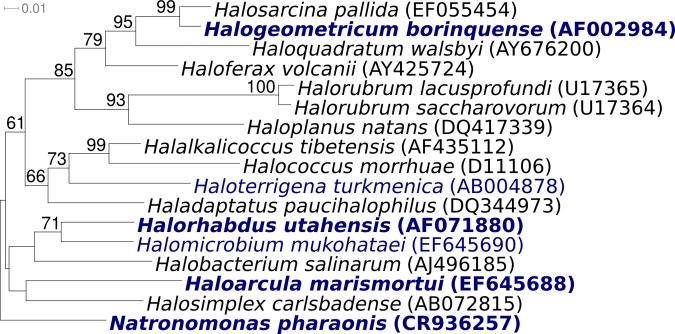
Phylogenetic tree of *H. borinquense* PR3^T^ with a selection of type strains of the family *Halobacteriaceae*, inferred from 1,433 aligned 16S rRNA characters [2] under the maximum likelihood criterion [3,4]. The tree was rooted with *Natronomonas pharaonsis*, the deepest branching member of the family *Halobacteriaceae*. The branches are scaled in terms of the expected number of substitutions per site. Numbers above branches are support values from 1,000 bootstrap replicates. Strains with a genome sequencing project registered in GOLD [5] are printed in blue; published genomes in bold.

Strain PR3^T^ (= DSM 11551 = ATCC 700274 = JCM 10706) is the type strain of *Halogeometricum borinquense*, representing the sole species of the genus *Halogeometricum* [1]. Strain PR3^T^ was first described by Montalvo-Rodríguez *et al*. in 1998 [1] as Gram-stain negative and motile. The organism is of interest because of its position in the tree of life, where it is located between members of the *Haloferax/Halorubrum* cluster within the large euryarchaeal family *Halobacteraceae*(Figure 1). Here we present a summary classification and a set of features for *H. geometricum* PR3^T^ together with the description of the complete genomic sequencing and annotation.

## Classification and features

In addition to the solar salterns of Cabo Rojo, Puerto Rico, where the type strain PR3^T^ and two accompanying strains (PR7 and PR9) were initially isolated [1], the occurrence of strains or phylotypes closely related or belonging to *H. borinquense* have so far only been reported from high salt environments such as an Australian crystallizer pond [6], Maras Salterns in the Peruvian Andes [7], a salt field at Nie, Ishikawa Prefecture, Japan [8], the salterns of Tamilnadu, India (Kannan *et al*. unpublished), Exportadora del Sal, Guerro Negro, Mexico (FJ609942), a Taiwanese saltern soil (FJ348412), and a low-salt, sulfide- and sulfur-rich spring in southwestern Oklahoma, USA [9].

**Figure 2:**
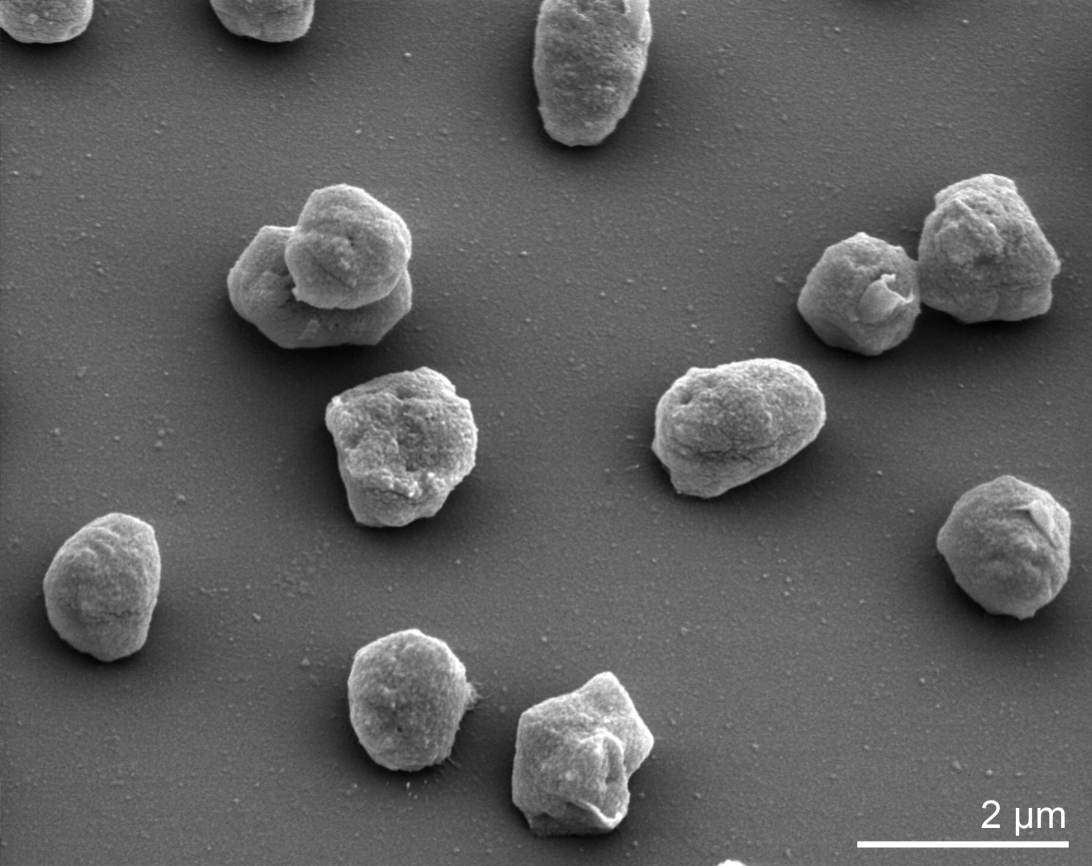
Scanning electron micrograph of *H. borinquense* PR3^T^ (Manfred Rohde, Helmholtz Centre for Infection Research, Braunschweig)

*H. geometricum* PR3^T^ cells are highly pleomorphic (short and long rods, squares, triangles and ovals) and motile by peritrichous flagella (Table 1 and Figure 2). Cells lyse in distilled water. Gas vesicles are present and are responsible for modifying the color of colonies or cell suspensions from red to pink. *H. geometricum* PR3^T^ is aerobic, but also capable of anaerobic growth with nitrate. No anaerobic growth on arginine (arginine dihydrolase is not present). At least 8% NaCl (w/v) is required for growth, reflecting the primary characteristic requirement for high salt concentrations of the *Halobacteriaceae* [18]. The optimal NaCl concentration range is 20-25***%*** NaCl (w/v) at 40°C (optimal growth temperature). Nitrate is reduced to nitrite with the production of gas [1]. Spores or other resting stages have not been reported [1].

**Table 1:** Classification and general features of *H. borinquense* PR3^T^ according to the MIGS recommendations [10]

**MIGS ID**	**Property**	**Term**	**Evidence code**
	Current classification	Domain *Archaea*	TAS [11]
		Phylum *Euryarchaeota*	TAS [12]
		Class *Halobacteria*	TAS [13]
		Order *Halobacteriales*	TAS [14]
		Family *Halobacteriaceae*	TAS [15]
		Genus *Halogeometricum*	TAS [1]
		Species *Halogeometricum borquinense*	TAS [1]
		Type strain PR3	TAS [1]
	Gram stain	negative	TAS [1]
	Cell shape	highly pleomorphic	TAS [1]
	Motility	motile	TAS [1]
	Sporulation	non-sporulating	NAS
	Temperature range	mesophile, between 22°C and 50°C	TAS [1]
	Optimum temperature	40°C	TAS [1]
	Salinity	halophile, at least 8% (w/v) NaCl	TAS [1]
MIGS-22	Oxygen requirement	primarily aerobic; facultatively anaerobic growth *via* nitrate reduction	TAS [1]
	Carbon source	glucose, mannose, fructose, xylose, maltose, trehalose, cellobiose, raffinose, glycerol	TAS [1]
	Energy source	carbohydrates	TAS [1]
MIGS-6	Habitat	aquatic	TAS [1]
MIGS-15	Biotic relationship	free living	NAS
MIGS-14	Pathogenicity	none	NAS
	Biosafety level	1	TAS [16]
	Isolation	solar salterns of Cabo Rojo, Puerto Rico	TAS [1]
MIGS-4	Geographic location	Cabo Rojo, Puerto Rico	TAS [1]
MIGS-5	Sample collection time	1994	TAS [1]
MIGS-4.1 MIGS-4.2	Latitude / Longitude	18,088 / -67,147	TAS [1]
MIGS-4.3	Depth	not reported	
MIGS-4.4	Altitude	sea level	NAS

Evidence codes - IDA: Inferred from Direct Assay (first time in publication); TAS: Traceable Author Statement (i.e., a direct report exists in the literature); NAS: Non-traceable Author Statement (i.e., not directly observed for the living, isolated sample, but based on a generally accepted property for the species, or anecdotal evidence). These evidence codes are from the Gene Ontology project [17]. If the evidence code is IDA then the property was directly observed for a living isolate by one of the authors or an expert mentioned in the acknowledgements.

*H. geometricum* PR3^T^ is capable of degrading gelatin, but starch is not hydrolysed. A number of sugars and polyols are used as carbon sources, and acid is produced from some sugars [1].

Figure 1 shows the phylogenetic neighborhood of *H. borinquense* strain PR3^T^ in a 16S rRNA based tree. Analysis of the two 16S rRNA gene sequences in the genome of strain PR3^T^ indicated that the two genes differ by five nucleotides (nts) from each other, and by 3-5 nts from the previously published 16S rRNA sequence generated from DSM 11551 (AF002984). The slight differences between the genome data and the reported 16S rRNA gene sequence are most likely the result of sequencing errors in the previously reported sequence data.

The quinone composition of *H. borinquense* strain PR3^T^ has not been recorded, but based on reports from other members of the family *Halobacteriaceae* menaquinones with eight isoprenoid units are likely to be present. Typically both MK-8 and MK-8 (VIII-H_2_) are predicted. The lipids are based on isoprenoid diether lipids, but the exact nature of the isoprenoid side chains remains to be investigated. The major phospholipids are the diether, isoprenoid analogs of phosphatidylglycerol and methyl-phosphatidylglycerophosphate (typical of all members of the family *Halobacteriaceae*), the diether analog of phosphatidyl-glycerol sulfate is absent [1]. A single glycolipid has been reported with an R_f_ value similar to that of the triglycosyl diether from *Haloarcula marismortui*, but its structure has not been determined [1]. The pigments responsible for the red color of the cells have not been determined, but it may be predicted that they are carotenoids, probably bacterioruberins. Outer cell layers are probably proteinaceous. The presence of peptidoglycan has not been investigated, but is generally absent from members of this family *Halobacteriaceae.*

## Genome sequencing and annotation

### Genome project history

This organism was selected for sequencing on the basis of each phylogenetic position, and is part of the *** G****enomic* *** E****ncyclopedia of* *** B****acteria and* *** A****rchaea * project. The genome project is deposited in the Genome OnLine Database [5]. The complete genome sequence has not yet been released from GenBank. Sequencing, finishing and annotation were performed by the DOE Joint Genome Institute (JGI). A summary of the project information is shown in Table 2.

**Table 2:** Genome sequencing project information

**MIGS ID**	**Property**	**Term**
MIGS-31	Finishing quality	Finished
MIGS-28	Libraries used	Two genomic libraries: 8kb pMCL200 and fosmid pcc1Fos Sanger libraries. One 454 pyrosequence standard library.
MIGS-29	Sequencing platforms	ABI3730, 454 GS FLX
MIGS-31.2	Sequencing coverage	9.7× Sanger; 21.8× pyrosequencing
MIGS-30	Assemblers	Newbler, PGA
MIGS-32	Gene calling method	GeneMark 4.6b, tRNAScan-SE-1.23, infernal 0.81
	INSDC / Genbank ID	CP001688
	Genbank Date of Release	September 10, 2009
	GOLD ID	Gc01108
	NCBI project ID	20743
	Database: IMG-GEBA	2501416934
MIGS-13	Source material identifier	DSM 11551
	Project relevance	Tree of Life, GEBA

### Growth conditions and DNA isolation

*H. borinquense* PR3^T^, DSM 11551, was grown in DSMZ medium 372 (*Halobacteria* Medium) at 35°C [19]. DNA was isolated from 1-1.5 g of cell paste using a Qiagen Genomic 500 DNA Kit (Qiagen, Hilden, Germany) with a modified protocol for cell lysis, LALMP procedure according to Wu *et al*. [20]..

### Genome sequencing and assembly

The genome was sequenced using a combination of Sanger and 454 sequencing platforms. All general aspects of library construction and sequencing performed at the JGI can be found at http://www.jgi.doe.gov/. 454 Pyrosequencing reads were assembled using the Newbler assembler version v 2.0.0 (Roche). Large Newbler contigs were broken into 4,435 overlapping fragments of 1,000 bp and entered into assembly as pseudo-reads. The sequences were assigned quality scores based on Newbler consensus q-scores with modifications to account for overlap redundancy and adjust inflated q-scores. A hybrid 454/Sanger assembly was made using the PGA assembler. Possible mis-assemblies were corrected and gaps between contigs were closed by custom primer walks from sub-clones or PCR products. A total of 2,826 Sanger finishing reads were produced. The error rate of the completed genome sequence is less than 1 in 100,000. Together all sequence types provided 31.5× coverage of the genome.

### Genome annotation

Genes were identified using GeneMark [21] as part of the genome annotation pipeline in the Integrated Microbial Genomes Expert Review (IMG-ER) system [22], followed by a round of manual curation using the JGI GenePRIMP pipeline [23]. The predicted CDSs were translated and used to search the National Center for Biotechnology Information (NCBI) nonredundant database, UniProt, TIGRFam, Pfam, PRIAM, KEGG, COG, and InterPro databases. The tRNAScanSE tool [24] was used to find tRNA genes, whereas ribosomal RNAs were found by using the tool RNAmmer [25]. Other non coding RNAs were identified by searching the genome for the Rfam profiles using INFERNAL (v0.81) [26]. Additional gene prediction analysis and manual functional annotation was performed within the Integrated Microbial Genomes (IMG) platform [27].

### Metabolic network analysis

The metabolic Pathway/Genome Database (PGDB) was computationally generated using Pathway Tools software version 12.5 [28] and MetaCyc version 12.5 [29], based on annotated EC numbers and a customized enzyme name mapping file. It has undergone no subsequent manual curation and may contain errors, similar to a Tier 3 BioCyc PGDB [30].

### Genome properties

**Figure 3:**
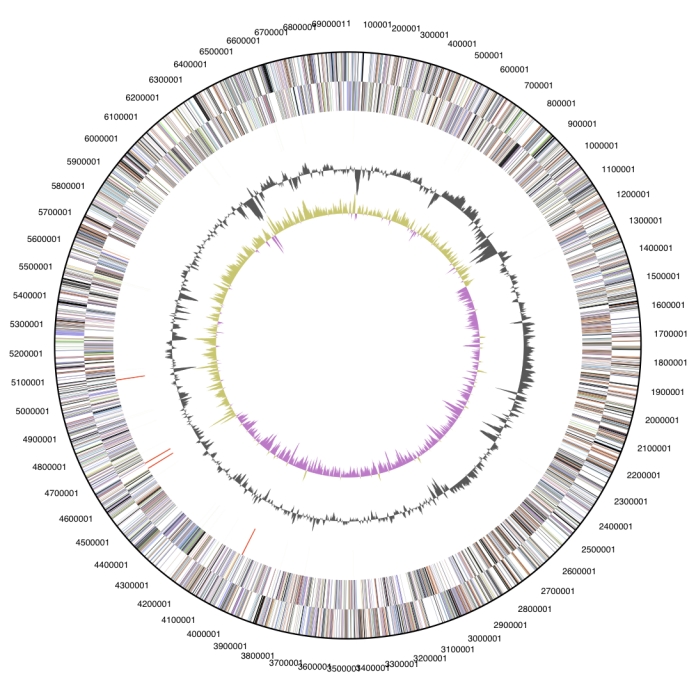
Graphical circular map of the genome. From outside to the center: Genes on forward strand (color by COG categories), Genes on reverse strand (color by COG categories), RNA genes (tRNAs green, rRNAs red, other RNAs black), GC content, GC skew.

**Figure 4:**
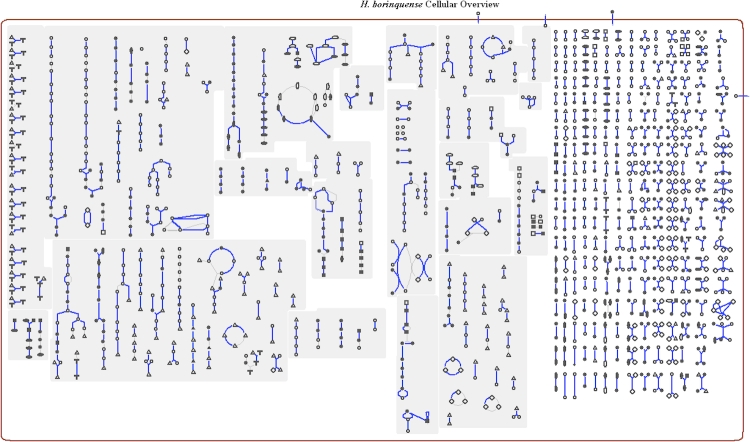
Schematic cellular overview diagram of all pathways of *H. borinquense* strain PR3^T^. Nodes represent metabolites, with shape indicating class of metabolite. Lines represent reactions.

The genome is 3,944,467 bp long and comprises one main circular chromosome with a 60% GC content and five plasmids. Of the 3,994 genes predicted, 3,937 were protein coding genes, and 57 RNAs. Thirty seven pseudogenes  were also identified. A total of 62% of the genes were assigned a putative function while the remaining ones are annotated as hypothetical proteins. The properties and the statistics of the genome are summarized in Table 3. The distribution of genes into COGs functional categories is presented in Figure 3 and Table 4. A cellular overview diagram is presented in Figure 4, followed by a summary of metabolic network statistics shown in Table 5.

**Table 3:** Genome Statistics

**Attribute**	**Value**	**% of Total**
Genome size (bp)	3,944,467	100.00%
DNA Coding region (bp)	3,441,571	87.25%
DNA G+C content (bp)	2,364,339	59.94%
Number of replicons	1	
Extrachromosomal elements	5	
Total genes	3994	100.00%
RNA genes	57	1.90%
rRNA operons	2	
Protein-coding genes	3937	98.57%
Pseudogenes	37	0.93%
Genes with function prediction	2486	62.24%
Genes in paralog clusters	741	18.55%
Genes assigned to COGs	2449	61.32%
Genes assigned Pfam domains	2385	59.71%
Genes with signal peptides	533	13.35%
Genes with transmembrane helices	971	24.31%
CRISPR repeats	1	

**Table 4:** Number of genes associated with the general COG functional categories

**Code**	**Value**	**% age**	**Description**
J	162	4.1	Translation, ribosomal structure and biogenesis
A	1	0.0	RNA processing and modification
K	140	3.6	Transcription
L	138	3.5	Replication, recombination and repair
B	3	0.0	Chromatin structure and dynamics
D	0	0.1	Cell cycle control, mitosis and meiosis
Y	0	0.0	Nuclear structure
V	46	1.2	Defense mechanisms
T	113	2.8	Signal transduction mechanisms
M	87	2.2	Cell wall/membrane biogenesis
N	38	0.1	Cell motility
Z	0	0.0	Cytoskeleton
W	0	0.0	Extracellular structures
U	27	0.7	Intracellular trafficking and secretion
O	123	3.1	Posttranslational modification, protein turnover, chaperones
C	174	4.4	Energy production and conversion
G	124	3.1	Carbohydrate transport and metabolism
E	271	6.9	Amino acid transport and metabolism
F	77	1.9	Nucleotide transport and metabolism
H	140	3.5	Coenzyme transport and metabolism
I	98	2.5	Lipid transport and metabolism
P	178	4.5	Inorganic ion transport and metabolism
Q	60	1.5	Secondary metabolites biosynthesis, transport and catabolism
R	433	11.0	General function prediction only
S	227	5.8	Function unknown
-	1488	37.8	Not in COGs

**Table 5:** Metabolic Network Statistics

**Attribute**	Value
Total genes	**3801**
Enzymes	578
Enzymatic reactions	687
Metabolic pathways	125
Metabolites	578
